# Serum Iron Levels and Copper-to-Zinc Ratio in Sickle Cell Disease

**DOI:** 10.3390/medicina55050180

**Published:** 2019-05-21

**Authors:** Charles Antwi-Boasiako, Gifty B. Dankwah, Robert Aryee, Charles Hayfron-Benjamin, Alfred Doku, Benoit Banga N’guessan, Isaac Julius Asiedu-Gyekye, Andrew D. Campbell

**Affiliations:** 1 Department of Physiology, School of Biomedical and Allied Health Sciences, University of Ghana, Accra 00233, Ghana; gdankwah@gmail.com (G.B.D.); bobby200055@gmail.com (R.A.); charlesfhb1@gmail.com (C.H.-B.); 2 Departments of Anaesthesia, School of Medicine and Dentistry, University of Ghana, Accra 00233, Ghana; 3 Department of Internal Medicine, School of Medicine and Dentistry, University of Ghana, Accra 00233, Ghana; dokukavin@gmail.com; 4 Department of Pharmacology and Toxicology, School of Pharmacy, University of Ghana, Accra 00233, Ghana; benoitnguessan@yahoo.com (B.B.N.); asiedugyekye@yahoo.co.uk (I.J.A.-G.); 5 Center for Cancer and Blood Disorders Children’s National Medical Center George Washington University School of Medicine and Health Sciences, Washington, DC 20052, USA; acampbell@childrensnational.org

**Keywords:** copper, zinc, iron, homeostasis, oxidative stress, sickle cell disease

## Abstract

*Background and Objectives*: Altered copper and zinc homeostasis may influence the antioxidant defense system and consequently lead to oxidative stress and associated complications in sickle cell disease (SCD) patients. Iron levels have been reported to increase in sickle cell patients due to frequent blood transfusion, chronic intravenous haemolysis and increased absorption of iron from the gastrointestinal tract. These elevated levels of iron may also lead to extensive oxidative damage. The current study evaluated serum levels of iron, copper and zinc in SCD patients and “healthy” controls. *Materials and Methods*: The study was a cross-sectional one, comprising 90 SCD patients with Haemoglobin SS and Haemoglobin SC genotypes and 50 HbAA “healthy” controls. Serum levels of iron, copper and zinc were measured using a Flame Atomic Absorption Spectrometer (Variant 240FS manufactured by VARIAN Australia Pty Ltd, VIC, Australia). Copper and zinc ratios were calculated and analyzed. *Results*: Serum levels of iron and copper were significantly elevated in the SCD patients, compared to their “healthy” counterparts (*p* < 0.001). These levels were further increased in patients with haemoglobin SS in vaso-occlusive crises (HbSS VOCs). Serum zinc levels were, however, significantly lower in the SCD patients, particularly during vaso-occlusion. The copper-to-zinc ratio was also found to be significantly higher in the SCD patients. *Conclusion*: Elevated copper-to-zinc ratio may be a biomarker of sickle cell oxidative stress and associated complications. The ratio may also be informative for the management of sickle cell oxidative burden. The significantly lower levels of zinc in the SCD patients may warrant zinc supplementation.

## 1. Introduction

Sickle cell disease (SCD) is a common inherited haemoglobinopathy in sub-Saharan Africa, caused by abnormal haemoglobin (HbS). The commonest clinical phenotype, which is termed sickle cell anaemia, is the homozygous form (HbSS). Heterozygous forms including HbSC arise from another variant haemoglobin gene (HbC) in addition to the HbS [1]. Sickle cell patients are characterized by frequent vaso-occlusive crises and chronic intravascular haemolysis [2]. This leads to increased oxidative stress, and contributes to inflammation in sickle cell patients [3]. The disease, with high prevalence in Ghana [4], is also associated with high risk of essential micronutrient deficiencies, including zinc [5]. An imbalance of oxidants/antioxidants in sickle cell patients may lead to the destruction of DNA, proteins and lipids [6]. Consequently, oxidative burden may significantly increase in such patients, leading to several other chronic complications.

Iron released from haemoglobin can be oxidized by H_2_O_2_ to form hydroxyl radicals by the Fenton’s reaction, which increases the levels of reactive oxygen species (ROS), resulting in protein oxidation, lipid peroxidation, and damage to cellular macromolecules, such as DNA and mitochondrial dysfunction [6,7,8]. Thus, elevated levels of iron may increase the oxidative burden in sickle cell patients. Zinc deficiency in sickle cell patients was reported several years ago [9] and was associated with several clinical manifestations, such as poor growth and delayed wound healing [10]. Copper has also been reported to be very essential for the catalytic activity of certain enzymes. Zinc and copper are required for full activity of superoxide dismutase [11].

An imbalanced homeostasis of copper and zinc, as well as an imbalance of their ratio (Cu/Zn ratio), has been reported to be associated with the pathophysiology of certain diseases [12,13] including SCD [14] elsewhere. The relative serum levels of iron, copper and zinc implicated in the pathophysiology of SCD have not been evaluated in Ghanaian sickle cell patients. The current study, therefore, evaluated serum iron, copper and zinc levels in Haemoglobin SS, Haemoglobin SC and Haemoglobin AA controls as potential biomarkers of sickle cell oxidative burden.

## 2. Materials and Methods

### 2.1. Study Site

The study was conducted at the Korle-Bu Teaching Hospital (KBTH) in the Greater Accra Region of Ghana. This is a tertiary hospital where most cases are referred to for treatment. The Sickle Cell Unit, which is the largest tertiary clinic for SCD in Ghana, is one of several units of the hospital. The controls were taken from the Accra Area Blood Centre located at the KBTH.

### 2.2. Study Design, Subject Recruitment, and Data Collection

This was a cross-sectional study involving 90 SCD patients and 50 controls at the KBTH from January–April, 2018. The study participants were recruited from the Sickle cell unit of the KBTH who came for their routine hospital examination. Clinically diagnosed SCD patients who met the inclusion criteria and consented to the study were recruited. SCD patients were genotyped using haemoglobin electrophoresis. SCD patients recruited were those with vaso-occlusive crises and at steady state. Steady state was clinically defined as a patient who has been well and had not been in crisis for at least 2 weeks and went about his activities. Vaso-occlusive crisis was also defined as pains in the bones, muscles and joints, which could not be attributed to any other cause. Vaso-occlusive crisis VOC cases were diagnosed by the physician on duty. Patients with conditions such as diabetes, renal disease, gastrointestinal disease or coronary artery disease were excluded from the study. Patients who had received transfused blood 3 months before the study were also not included in the study. The “healthy” controls were recruited from the Accra Area Blood Transfusion at the KBTH.

### 2.3. Laboratory Analysis

Approximately 5 mL of venous blood was drawn with a 19G hypodermic needle fixed on a 5 mL syringe from patients and controls into plain tubes. Sera were prepared from the whole blood, by centrifugation at 3000 rpm for 15 min, and kept at −20 °C for determination of iron, copper and zinc. A Flame Atomic Absorption Spectrometer (Variant 240FS manufactured by VARIAN Australia Pty Ltd, VIC, Australia) was used to measure the levels of iron, copper and zinc [15].

### 2.4. Data Analysis

The data were expressed as mean ± standard deviation and analyzed using SPSS version 20 software (SPSS Inc., Chicago, IL, USA). The analysis of variance was used to compare means between more than two groups, followed by Turkey’s post hoc test. Spearman’s correlation was used to determine the relationship between the copper-to zinc ratio and haemoglobin levels among the study subjects. A *p*-value of less than 0.05 was considered statistically significant.

### 2.5. Ethics Statement

Ethical approval for the study was obtained from the Ethical and Protocol Review Committee of the College of Health Sciences, University of Ghana on 30 March 2017 and was valid until 30 April 2018, with protocol identification number CHS-Et/M.8-P 3.2/2016–2017. The study participants consented before partaking in the study.

## 3. Results

### 3.1. Demographic Characteristics of the Study Participants

The 140 participants enrolled in this study were made up of 90 SCD patients and 50 “healthy” controls (HbAA). The SCD patients were comprised of 43 males and 47 females while the controls were made up of 20 males and 30 females. The ratio of HbSS steady state and HbSC steady state patients was 1:1. The ration of patients with HbSS VOC and HbSC VOC was also 1:1 (Table 1). There was no significant difference in the male and female distribution (*p* = 0.4074).

### 3.2. Serum Levels of Iron, Copper and Zinc in the Study Participants

The serum levels of iron were significantly higher in the SCD patients compared to the “healthy” controls (*p* < 0.001). Serum iron levels were further increased significantly in patients with HbSS VOC (*p* < 0.001). Overall, patients with the HbSS genotype had relatively higher serum levels of iron in this study. Sickle cell disease patients generally had higher levels of copper compared to the HbAA controls. The differences in levels of serum copper among the study subjects were significant (*p* < 0.001). Again, it was observed that SCD patients with the HbSS genotype had significantly higher levels of copper. Although serum levels of copper were higher in patients with HbSC VOC, compared to the HbSC steady state, the difference was not significant (*p* = 0.087). Among the study participants, serum zinc levels were significantly higher in the HbAA controls (*p* < 0.001). The levels were further reduced in patients with the HbSS genotype, particularly among those with vaso-occlusive crises. As expected, SCD patients generally had a significantly higher copper-to-zinc ratio compared to the HbAA controls (*p* < 0.001). The ratios were even elevated in all the HbSS patients. The higher copper-to-zinc ratio was, however, observed in patients with HbSS VOC among the SCD patients. Haemoglobin levels were significantly reduced in the SCD patients compared to the controls (*p* < 0.001). Of the SCD patients, the lowest haemoglobin level was recorded in patients with HbSS VOC (Table 2).

### 3.3. Correlation between Copper-to-Zinc Ratio and Haemoglobin Among the Study Participants

**Figure 1:**
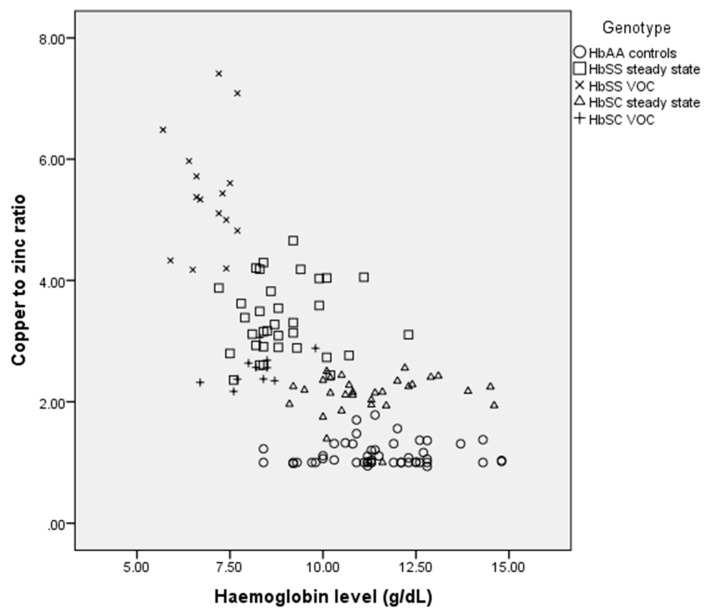
Correlation of Copper-to-zinc ratio and haemoglobin levels in the study participants.

Spearman’s correlation showed that the copper-to-zinc ratio correlated significantly with levels of haemoglobin in some of the study participants. Although copper-to-zinc ratios were higher in all the sickle cell patients compared to their “healthy” counterparts, there was no correlation with levels of haemoglobin in the HbSS and HbSS VOC patients. Meanwhile, the haemoglobin levels of the SCD patients were lower. The Spearman’s correlations and the p-values for the HbAA controls, HbSC steady state, HbSS steady state, HbSC VOC and HbSS VOC are r = 0.080, *p* = 0.587; r = 0.134, *p* = 0.480; r = 0.018, *p* = 0.920; r = 0.506, *p* = 0.113; r = −0.027, *p* = 0.924, respectively (Figure 1).

## 4. Discussion

In this present study, serum iron and copper levels, which contribute to oxidative stress in haemoglobinopathies such as SCD, were significantly higher in the cases compared to the controls. This is in line with other similar studies [14,16], which also reported increased levels of serum iron in patients with sickle cell anaemia, compared to the control group. Iron is a powerful oxidant which can convert hydrogen peroxide to free radicals. These free radicals, when produced, could cause oxidative damage to cells by attacking lipids, proteins and DNA [17]. Thus, a higher level of iron is detrimental to cells and results in extensive oxidative damage. The higher levels of serum iron in the HbSS VOC patients could partly be a result of the rapid auto-oxidation of the HbS molecule as well as the severe intravascular haemolysis observed in such patients. Thus, it seems likely that the oxidative stress in patients with HbSS VOC may be higher than those with HbSC VOC.

Copper, which functions as an antioxidant, is another cofactor found in many enzymes. It may however act as a pro-oxidant when present in high concentrations [18]. The high levels of serum copper in the SCD patients suggest in part that they promote free radicals, leading to harmful effects [19] often observed in sickle cell patients generally. Previous studies have also reported elevated levels of serum copper in sickle cell patients [14,16,19,20]. The higher level of serum copper in the HbSS VOC patients is partly due to the chronic haemolytic condition often observed in patients with vaso-occlusion. Osredkar and Sustar discovered in their study that copper levels in the body are influenced by zinc bioavailability. They observed that zinc deficiency or low zinc levels as seen in the SCD patients significantly increased the absorption of copper in the gastrointestinal tract [13]. Therefore, in sickle cell patients with impaired zinc bioavailability, the level of copper is expected to increase.

The level of serum zinc was significantly lower in the SCD patients with the HbSS genotype, especially during VOC. Zinc deficiency has been well documented in patients with SCD [8,9,20]. Findings from this current study agree with previous results on the levels of serum zinc in sickle cell patients [14,16,19,21]. Zinc deficiency has also been previously noted in SCD in other studies [22]. The lower serum levels of zinc in SCD is partly due to factors including reduced reabsorption of zinc in the renal tubules due to sickling and disturbed metabolism of zinc metalloenzymes in SCD [10], leading to a possible hyperzincuria [23]. Zinc is an important element in the body and a cofactor for many distinct metalloenzymes which perform a wide range of functions [24]. This includes synthesis of various biomolecules, and zinc also plays important roles in cellular growth, differentiation and metabolism [24]. The presence of zinc at the required level in the body is, therefore, beneficial to the SCD patient and has been reported to decrease the incidence of infections in SCD. This may consequently contribute to reduced oxidative damage [25]. The reduced zinc bioavailability in sickle cell patients may consequently lead to several complications, such as growth retardation and delayed wound healing [10]. Therefore, zinc supplementation may be required in sickle cell patients, and factors that affect zinc availability in sickle cell patients should be rectified to improve management of sickle cell patients.

The copper to zinc ratio has been used as a predictive measure of several clinical complications [26,27,28] including sickle cell [14]. By comparison, a similar study reported that copper to zinc ratios are significantly higher in sickle cell patients [14]. The lower levels of serum zinc in sickle cell patients accounted for this high ratio. The ratio was even higher in patients with HbSS in the vaso-occlusive crises. This may partly be a result of frequent inflammation and associated intravascular haemolysis in these patients. Thus, the copper to zinc ratio could give a better predictive diagnosis of oxidative stress in these patients than copper or zinc status alone [12,29]. It is worth mentioning that the main limitation of this study was our relatively small sample size. Moreover, we could not assess the severity of the disease among the patients. Ferritin, which is a storage medium of iron, could have been a better parameter for iron homeostasis. However, the levels of serum ferritin were not determined in this current study.

## 5. Conclusions

Serum iron levels were significantly higher in the SCD patients, especially those with the HbSS genotype compared to the HbAA controls in the present study. The levels were further elevated in the HbSS VOC patients. The SCD patients also had significantly higher serum Cu/Zn ratio, which may be a promising marker for oxidative stress in sickle cell patients.

## Figures and Tables

**Table 1:** Demographic features of the study participants.

Parameter	Genotype				
	HbAA (*n* = 50)	HbSC Steady State (*n* = 30)	HbSS Steady State (*n* = 34)	HbSC VOC (*n* = 11)	HbSS VOC (*n* = 15)
Mean Age	32.8 ± 10.4 years	38.2 ± 15.1 years	25.0 ± 9.0 years	23.3 ± 9.5 years	21.3 ± 4.6 years
Gender					
Male n (%)	20 (40.0)	13 (43.3)	20 (58.8)	5 (45.5)	5 (33.3)
Female n (%)	30 (60.0)	17 (56.7)	14 (41.2)	6 (54.5)	10 (66.7)

Values are expressed as mean ± standard deviation (SD).

**Table 2:** Mean Serum levels of co-factors in controls and sickle cell disease (SCD) patients.

Parameter	Genotype					
	HbAA (*n* = 50)	HbSC Steady State (*n* = 30)	HbSS Steady State (*n* = 34)	HbSC VOC (*n* = 11)	HbSS VOC (*n* = 15)	*p*-Value
Iron (µg/dL)	106.0 ± 12.7	123.8 ± 6.1	164.3 ± 7.2	139.3 ± 6.9	175.9 ± 5.3	<0.001
Copper (µg/dL)	114.0 ± 16.3	179 ± 23.9	220.9 ± 27.8	194.6 ± 12.6	277.9 ± 41.7	<0.001
Zinc (µg/dL)	101.4 ± 9.4	85.0 ± 6.9	66.5 ± 5.8	76.9 ± 5.2	51.3 ± 6.0	<0.001
Copper/Zinc ratio	1.1 ± 0.2	2.1 ± 0.3	3.4 ± 0.6	2.5 ± 0.3	5.5 ± 1.0	<0.001
Haemoglobin (g/dL)	11.5 ± 1.5	11.3 ± 1.5	8.9 ± 1.1	8.2 ± 0.8	6.9 ± 0.6	<0.001

Values are expressed as mean ± standard deviation (SD); significant at *p ≤* 0.05.
